# Reduction of inflammation and T cell activation after 6 months of cART initiation during acute, but not in early chronic HIV-1 infection

**DOI:** 10.1186/s12977-018-0458-6

**Published:** 2018-12-12

**Authors:** Hury Hellen Souza de Paula, Ana Cristina Garcia Ferreira, Diogo Gama Caetano, Edson Delatorre, Sylvia Lopes Maia Teixeira, Lara Esteves Coelho, Eduarda Grinsztejn João, Michelle Morata de Andrade, Sandra Wagner Cardoso, Beatriz Grinsztejn, Valdilea Gonçalves Veloso, Mariza Gonçalves Morgado, Monick Lindenmeyer Guimarães, Fernanda Heloise Côrtes

**Affiliations:** 1 Laboratório de Aids e Imunologia Molecular, Instituto Oswaldo Cruz –IOC, FIOCRUZ, Av Brasil, 4365, Pavilhão Leônidas Deane, sala 401, Rio de Janeiro, 21040360 Brazil; 2 Laboratório de Pesquisa Clínica em DST e Aids, Instituto Nacional de Infectologia Evandro Chagas - INI, FIOCRUZ, Rio de Janeiro, 25984-220 Brazil

**Keywords:** HIV-1 acute infection, Inflammation, cART, Immune activation, IP-10, IL-18

## Abstract

**Objectives:**

To investigate the impact of early combined antiretroviral therapy (cART) on inflammation biomarkers and immune activation during acute and early chronic HIV-1 infection.

**Methods:**

We included 12 acute (AHI), 11 early chronic (EcHI), and 18 late chronic HIV-1-infected (LcHI) individuals who were treated with cART and 18 HIV-1-uninfected (HIV-neg) individuals. Plasmatic levels of inflammation biomarkers, CD8^+^CD38^+^HLA-DR^+^ T cell frequencies, CD4 T cell counts, CD4/CD8 ratio, total HIV-1 DNA and plasmatic viral load were evaluated. Mann–Whitney test, Pearson and Spearman correlation, and linear regression models were used for statistical analyses.

**Results:**

IP-10, IL-18, and sCD163 were significantly elevated at pre-ART in the AHI and EcHI groups, showing a significant reduction after 6 months of cART in the AHI group, achieving similar levels to the HIV-neg group. For the EcHI group, the IP-10 and sCD163 levels were also significantly reduced on M6-ART; however, IP-10 levels remained higher than in the HIV-neg group, and no significant reduction of IL-18 levels was observed. The CD8^+^ T cell activation levels were elevated in the AHI and EcHI groups at pre-ART and showed a significant reduction on M6-ART, but they were similar to levels seen for HIV-neg only after 12 months of cART. At pre-ART, IP-10 levels but not IL-18 levels were positively correlated with HIV-1 viral load in the AHI group.

**Conclusions:**

Early initiation of cART in HIV infection can reduce systemic inflammation, but the earlier normalization of the inflammation markers was only observed when cART was initiated in the acute phase of infection. A slower dynamic of reduction was observed for CD8^+^ T cell activation.

**Electronic supplementary material:**

The online version of this article (10.1186/s12977-018-0458-6) contains supplementary material, which is available to authorized users.

## Introduction

High systemic immune activation has a pivotal role in HIV-1 pathogenesis [1]. Studies have associated the elevated immune activation with CD4^+^ T cell depletion and progression to AIDS [2–7]. During primary HIV-1 infection (PHI), viral loads can reach values higher than one million HIV-1 RNA copies/mL, and a significant decrease in CD4^+^ T cell counts occurs [8, 9]. A high production of pro-inflammatory cytokines, called a “cytokine storm”, arises in response to virus replication [10]. A controlled initial pro-inflammatory immune response may be beneficial, as observed in natural SIV hosts, which displayed significant increases in plasma cytokines and interferon-stimulated gene (ISG) expression, normalized four weeks after infection [11]. However, in the context of HIV-1 infection, the pro-inflammatory response remains elevated after the end of the acute phase, and most individuals without cART develop progressive immune dysregulation, culminating in AIDS [8].

The cART has had an enormous impact on mortality and morbidity in HIV-1 infection [12–14]. Studies in the cART era have demonstrated a shift in the causes of death and morbidities in HIV-1-infected individuals, with a proportional increase of non-AIDS-related events, such as cardiovascular, liver, and renal diseases, when compared with AIDS-related events [15–20]. Also, a higher prevalence of age-related noninfectious comorbidities was observed in HIV-1-infected individuals than in the general population [21]. Alongside this, a strong association between inflammation and AIDS and non-AIDS-events became evident [22–24]. Several biomarkers of inflammation have been associated with disease progression and mortality in HIV-1 infection [25]. Among these markers, IL-6 and CRP were associated with an increased risk of AIDS [23] or death [22, 24]. IL-6 levels, at time of seroconversion, also predicted HIV-1 disease progression [26]. The markers of monocyte activation sCD14 and sCD163 are associated with a higher risk of death [27–29]. And, during the PHI, CRP levels were significantly higher in HIV-1 infected individuals than in HIV-1 uninfected individuals [5]. Other biomarkers elevated during PHI were IP-10 and IL-18, with an association between IP-10 and a faster disease progression [30, 31]. Continued IL-18 and sCD14 elevation were also associated with clinical cART failure [32].

Although cART reduces immune activation and inflammation [5, 22], these remain higher in HIV-1-infected individuals than in HIV-1-uninfected individuals, even after long-term viral suppression [33]. Studies have shown the benefits of initiation of cART during the acute or early infection [2–4, 6, 7]. The early therapy decreases immune system damage and the establishment of a large viral reservoir [6, 7]. HIV-1 infected patients who started cART during PHI presented an earlier immune reconstitution (CD4^+^ T cell count > 500 cells/mm^3^, CD4% > 30%, and CD4/CD8 ratio > 1) than cART in chronic patients [34]. Moreover, early cART decreases T cell activation [3, 7, 35] and markers of inflammation [3, 5]. Moreover, early ART is associated with a lower risk of development of non-AIDS morbidities [36, 37], and the study with the ANRS VISCONTI cohort demonstrated the possibility of a functional cure in individuals starting ART in primary infection [38].

In the present study, we compared the impact of cART on the levels of immunological markers between primary HIV-1 infected Brazilian individuals, who initiated cART, during the acute (Fiebig I-V) and early chronic phases (Fiebig VI). We evaluated CD4^+^ T cell counts, CD4/CD8 ratio, CD4^+^ and CD8^+^ T cell activation before (Pre-ART) and six (M6 ART) and 12 months (M12 ART) after cART initiation. We also evaluated plasmatic markers of inflammation and monocyte/macrophage activation at Pre-ART and M6 ART and total HIV-1 DNA at M6 and M12. We hypothesized that earlier cART would normalize levels of biomarkers associated with disease progression and death in HIV-1 infection.

## Materials and methods

### Study population and ethical statement

**Fig. 1:**
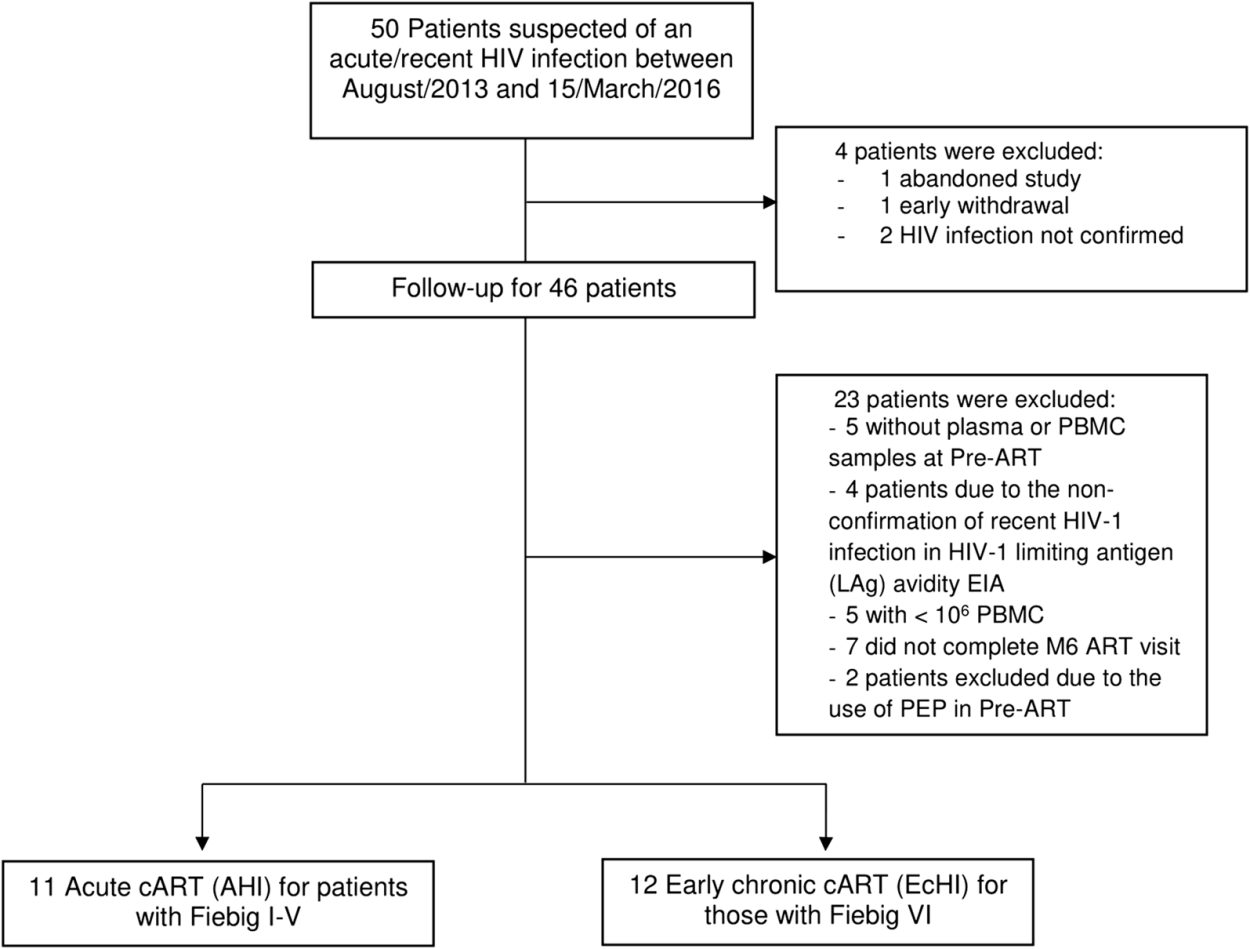
Flowchart for the selection of study participants

Study participants were recruited at the Instituto Nacional de Infectologia Evandro Chagas (INI), Rio de Janeiro, Brazil. The inclusion criteria include those who were over 18 years of age and with documented seroconversion within the previous 6 months. Recruitment occurred among those who sought INI or community-based out-of-health care units for HIV testing or care with HIV diagnostic tests suggesting acute HIV infection. Documentation of seroconversion could be the following: (a) a negative result for a third-generation HIV rapid test, followed by reactive HIV Ag/Ab combination assay, or a detectable HIV RNA on pooled HIV RNA testing subsequently confirmed with an individual HIV RNA test; (b) a reactive HIV serology and a documented HIV negative serology within the prior 6 months or a reactive western blot lacking p31 (pol) reactivity. The exclusion criteria were the following: lacking plasma or peripheral blood mononuclear cells (PBMC) on the baseline or M6 ART visits until March, 15th 2016 or less than 10 million cells on the PBMC samples. Of the fifty patients initially included, two did not confirm HIV infection, one abandoned the treatment, one withdrew from the study early, and 23 were excluded, as shown in Fig. 1; thus, the remaining 23 patients were included. The patients were categorized into Fiebig stages, as described in Fiebig et al. [39]. Briefly, Fiebig I was characterized by the presence of HIV-1 RNA in plasma samples (Abbott RealTime HIV-1) together with a fourth generation ELISA negative (Enzyme Linked Fluorescent Assay BioMérieux HIV DUO Ultra Assay); Fiebig II by a HIV-1 RNA detectable in plasma and the fourth generation ELISA with antigen positive and antibody negative; Fiebig III by a HIV-1 RNA detectable in plasma and a reactive HIV-1 antibody assay (by a 3rd generation assay with detection of IgG and/or IgM anti-HIV-1 or an antibody detection in the 4th generation BioMérieux ELISA HIV DUO Ultra Assay) but a Western Blot (Western Blot Cambridge Biotech HIV-1 negative (defined by the absence of HIV-1 specifc bands); Fiebig IV reactivity profile is identical to that present in the stage III, but with undetermined pattern in Western Blot (presence HIV-1 specific bands, but do not meet the criteria for the interpretation of reactive Western Blot, which is defined by the presence of two of the following three bands: p24, gp41 or gp120/160); Fiebig V reactivity profile is identical to that verified in stage IV, but with a reactive Western Blot result (defined by the presence of two of the following three bands:p24, gp41 or gp120/160); Fiebig VI by reactivity profile is identical to that observed in the stage V, but with the complete WB reactivity pattern, including the p31 band. After, they were divided into two groups: (a) starting cART in acute HIV-1 infection (AHI) for patients with Fiebig I-V (n = 11) and (b) starting cART in early chronic HIV-1 infection (EcHI) for those with Fiebig VI (n = 12). We also included late chronic treated HIV-1 infected patients (n = 18), with more than 5 years of HIV-1 viral load suppression by cART (LcHI), and HIV-1 uninfected individuals (HIV-neg, n = 18). All patients gave informed consent, and the INI Ethical Review Board approved this study.

### Sample collection and preparation

Blood samples were collected before cART initiation (Pre-ART), after six (M6 ART) and 12 months (M12 ART) of cART in EDTA and sodium heparin containing tubes. Peripheral blood mononuclear cells (PBMCs) were isolated from whole blood by Histopaque-1077 (Sigma-Aldrich, USA) density gradient centrifugation and kept in liquid nitrogen until use. Plasma aliquots were stored in a − 80  °C freezer until use.

### HIV-1 limiting antigen (LAg) avidity EIA

To confirm the recent HIV-1 infection status, EDTA-plasma specimens of all patients were subjected to a quantitative limiting antigen (LAg) avidity enzyme immunoassay (Sedia™ HIV-1 LAg-Avidity EIA, Sedia Biosciences Corporation, USA).

### CD4^+^ and CD8^+^ T cell count and plasma viral load determination

Absolute CD4^+^ and CD8^+^ T cell counts and %CD4 were obtained using the MultiTest TruCount-kit and the MultiSet software on a FACSCalibur flow cytometer (BD Biosciences, USA). Plasma HIV-1 viral loads were measured using the Abbott RealTime HIV-1 assay (Abbott Laboratories, Germany). For two AHI patients, data of CD4^+^ T cell counts were not available for the Pre-ART visit.

### Enzyme-Linked Immunosorbent Assay (ELISA)

The plasmatic concentrations of IP-10/CXCL10, IL-18, IL-6, sCD14, sCD163, and CRP were determined in EDTA-plasma samples by ELISA according to the manufacturer’s protocol at Pre-ART and M6 ART (R&D Systems, USA). For statistical analyses, points above the detection limit were replaced by the highest value in the assay. To evaluate in more detail the IP-10 and IL-18 levels in the AHI group, we also analyzed samples collected after 1, 2, and 3 months of cART initiation.

### Flow cytometry

Cryopreserved PBMCs from preART, M6 ART, and M12 ART were thawed and rested overnight. Then, the PBMCs were stained with anti-CD3 APC-H7, anti-CD4 PECF594, anti-CD8 APC, anti-CD38 PerCPCy5.5 and anti-HLA-DR PE to determine the percentage of activated CD4^+^ and CD8^+^ T cells by flow cytometry (all antibodies were purchased from BD Biosciences). The Fixable Viability Stain 450 (FVS 450-BD Biosciences, USA) was used to exclude nonviable cells. Samples were acquired using a BD FACSAria™ IIu flow cytometer (BD Biosciences, USA), and analyses were performed with FlowJo software v.10.0.7 (Tree Star, USA). For three EcHI and one AHI patients, PBMCs from Pre-ART presented viability below the levels accepted for testing.

### Quantification of HIV-1 total DNA in PBMCs

Total cellular DNA was extracted from 1 × 10^7^ cryopreserved PBMCs from Pre-ART and M12 ART visits using the QIAamp DNA Mini Kit (Qiagen, Germany). The Generic HIV^®^ DNA Cell Kit (Biocentric, France) was used to quantify the cell-associated HIV-1 DNA, following the manufacturer’s recommendations. The lower limit of detection was 40 HIV-1 DNA copies/10^6^ cells.

### HIV-1 subtyping

All HIV-1 *pol* (PR/RT) sequences were submitted to REGA HIV-1 Subtyping Tool. And them pure HIV-1 subtypes and circulating recombinant forms identified were confirm by Maximum-likelihood (ML) phylogenetic trees that were reconstructed with the PhyML 3.0 program [40] using the most appropriate nucleotide substitution model selected using program jModeltest v. 3.7 [41] and the approximate likelihood-ratio test (aLRT) was used to estimate the confidence of the branching on the tree. For unique recombinant forms we performed the bootscan analysis implemented in Simplot v3.5.1 software with the following parameters: 300nt window, 20nt increments, NJ method under Kimura’s two-parameter correction with 100 bootstrap replicates [42].

### Statistics

The Kruskal–Wallis test and Fisher’s exact test were used for the comparison of continuous variables and categorical variables, respectively, between groups. CD4^+^ T cell absolute counts and CD4/CD8 ratio, inflammatory and activation markers were analyzed using the Mann–Whitney test to compare variables between two groups and Wilcoxon matched pairs test to compare variables between Pre-ART, M6 ART and M12 ART intragroup. Correlations were performed using the Pearson test or Spearman test according the normality of the data. Linear regression models were used to evaluate factors (age and group) associated with cytokine levels at pre-ART and M6 ART. Statistical analyses were performed using GraphPad v6.0 (Prism Software, USA) and R software.

## Results

### Characteristics of the study population

Fifty-nine individuals were included in this study, divided into four groups: AHI, EcHI, LcHI, and HIV-neg (Table 1). The groups with PHI (AHI and EcHI) comprised the youngest individuals (median age 30.6 [interquartile range (IQR): 27.7, 39.5] for AHI, 28.3 [IQR 26.7, 29.9] for EcHI), and the LcHI group comprised the oldest individuals (median age 44.2 [IQR 40, 49.8]). We also found a different sex distribution among the groups; the HIV-neg group had 50% women, and the EcHI and AHI groups were composed exclusively men, all men who have sex with men (MSM). The HIV-1 viral load of the AHI group was significantly higher than that of the EcHI group at Pre-ART (median of 5.9 log [IQR 4.8, 6.5] in AHI, 4 log [IQR 3.7, 4.9] in EcHI and 1.6 log [IQR 1.6–1.6] in LcHI). After 6 months of cART, most individuals with PHI had undetectable viral load, except two EcHI (VL = 94 and 96 copies/mL) and one AHI (VL = 280 copies/mL). At 12 months of cART, all PHI subjects had an undetectable viral load, except one that has suppressed on M6 and presented a blip of 460 copies/mL at M12. The CD4^+^ T cell counts were higher in the HIV-neg group (median 831 cell/mm^3^ [IQR 757.2, 1129.5]) and the LcHI group (median 853 cell/mm^3^ [IQR 762, 983]) and lower in the EcHI group (median 566 cell/mm^3^ [IQR 389, 666.5]) and the AHI group (median 634 cell/mm^3^ [IQR 484.2, 885.5]). To EcHI and AHI individuals the HIV-1 subtypying was performed and the subtype B was the prevalent (Additional file 1: Table S1).

**Table 1:** Demographic and clinical characteristics of study participants

	HIV-neg (n = 18)	LcHI (n = 18)	EcHI (n = 11)	AHI (n = 12)	Total (n = 59)	*P* value
Age						0.001
Median (IQR)	37.1 (31.6; 49.3)	44.2 (40;49.8)	28.3 (26.7;29.9)	30.6 (27.7;39.5)	34.5 (27.9;46.5)	
Gender (%)						< 0.001
Female	9 (50)	7 (38.9)	0 (0)	0 (0)	16 (27.1)	
Male	9 (50)	11 (61.1)	11 (100)	12 (100)	43 (72.9)	
HIV-1 viral load						< 0.001
Log_10_, median (IQR)	NA	1.6 (1.6;1.6)	4 (3.7;4.9)	5.9 (4.8;6.5)	3.5 (1.6;4.9)	
CD4 + T cell count						0.001
Cells/mm^3^, median (IQR)	831 (757.2;1129.5)	853 (762;983)	566 (389;666.5)	634 (484.2;885.5)	784 (648;909)	

Analyses with age, HIV-1 viral load and CD4^+^ T cell count were performed using the Kruskal–Wallis

To EcHI and AHI we analyzed the pre-ART visit and for LcHI a pos-cART visit (median of 5 years after cART initiation)

Analyses with gender were performed using Fisher’s exact test

*LcHI* late chronic HIV-1 infection; *EcHI* early chronic HIV-1 infection; *AHI* acute HIV-1 infection; *NA* not applicable; *IQR* interquartile range

The avidity enzyme immunoassay results confirmed all AHI and EcHI individuals included in the study as recently HIV-1 infected, considering the window of 130 days established for this test.

### cART initiation during acute infection can normalize CD4^+^ T cells

**Fig. 2:**
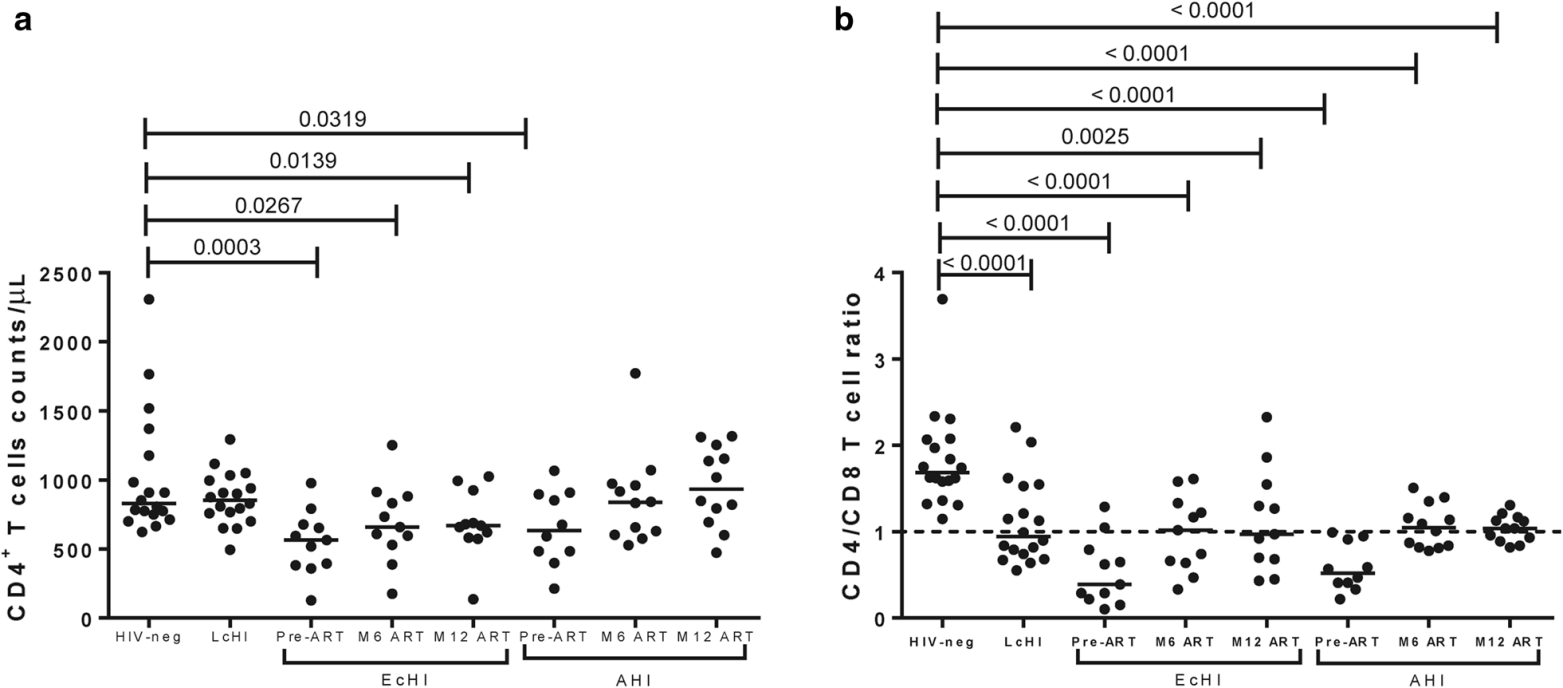
CD4^+^ T cell count and CD4/CD8 ratio on pre-ART and after six months. CD4^+^ T cell counts (**a**) and CD4/CD8 (**b**) ratios were evaluated using the MultiTest TruCount kit. *P* values were calculated using the Mann–Whitney test. All values < 0.05 were considered statistically significant. HIV-neg, HIV-1 uninfected individuals; LcHI, late chronic treated HIV-1 infection (median of 5 years after cART initiation); EcHI, early chronic HIV-1 infection; AHI, acute HIV-1 infection; M6 ART, six months after ART start; M12 ART, 12 months after ART start

We compared the CD4^+^ T cell absolute counts and the CD4/CD8 ratio between individuals who started cART in Fiebig I-V (AHI) and Fiebig VI stages (EcHI) with HIV-neg individuals (Fig. 2). The AHI group had CD4^+^ T cell counts (median = 838 cells/µL; IQR = 610–971) similar to the HIV-neg group (median = 831 cells/µL, IQR = 742–1227) 6 months after cART initiation, while the EcHI group, even after 12 months of cART (CD4 median = 670 cells/µL, IQR = 582–926), did not normalize CD4^+^ T cell counts (*P* = 0.0139) compared with the HIV-neg group (Fig. 2a). However, when we analyzed the CD4/CD8 ratio (Fig. 2b), both groups with recent HIV-1 infection presented lower CD4/CD8 ratios than the HIV-neg group after 6 and 12 months of cART. Moreover, we included a group of individuals who started cART in the late chronic phase (LcHI) and, despite a long time in cART with viral suppression and CD4^+^ T cell counts similar to the HIV-neg group, this group also presented a lower CD4/CD8 ratio than HIV-neg.

### IP-10 and IL-18 levels reduced after 6 months of cART in acute individuals

**Fig. 3:**
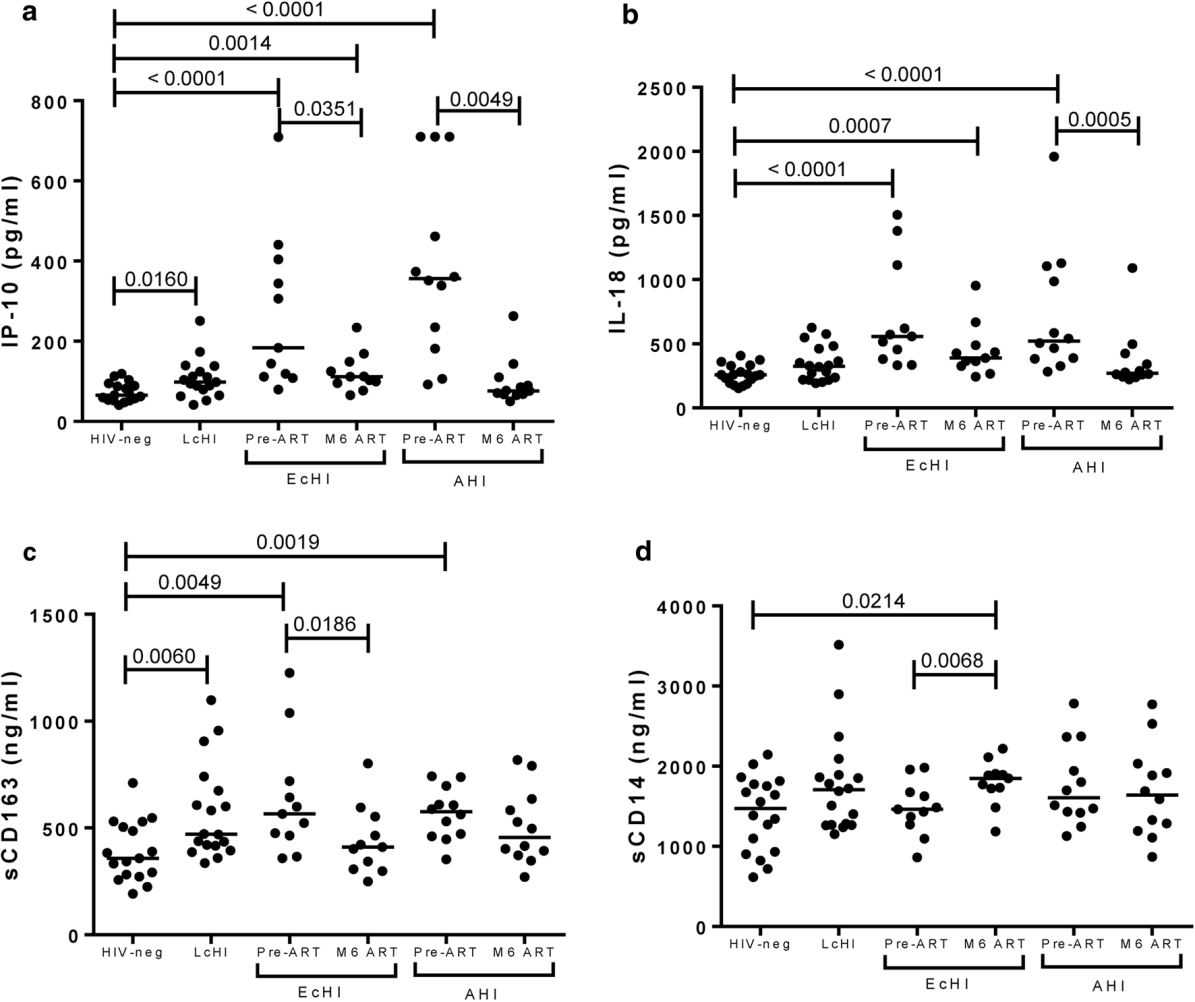
Plasmatic markers of inflammation on pre-ART and after 6 months. IP-10 (**a**), IL-18 (**b**), sCD163 (**c**), and sCD14 (**d**) were assessed by ELISA. *P* values were calculated using the Mann–Whitney test. All values < 0.05 were considered statistically significant. HIV-neg, HIV-1 uninfected individuals; LcHI, late chronic treated HIV-1 infection (median of 5 years after cART initiation); EcHI, early chronic HIV-1 infection; AHI, acute HIV-1 infection; M6 ART, 6 months after ART start; and M12 ART, 12 months after ART start

We evaluated six plasmatic markers of inflammation (IP-10/CXCL10, IL-18, IL-6, sCD14, sCD163, and CRP) before cART and 6 months after cART initiation (Fig. 3 and Additional file 1: Figure S1). These markers were chosen based in the following criteria: having a pro-inflammatory action, elevated levels in acute or chronic HIV-1 infection or being associated to AIDS progression or mortality [22, 25, 26, 28, 30, 31, 43]. IP-10 and IL-18 (*P* < 0.0001 for EcHI and AHI) and sCD163 (*P* = 0.0049 for EcHI and *P* = 0.0019 for AHI) were higher at pre-ART in both groups of PHI individuals than in the HIV-1 uninfected group. After 6 months of cART, IP-10 levels were significantly reduced in EcHI (*P* = 0.0351) and AHI (*P* = 0.0049) groups when compared with the pre-ART levels. However, only the AHI group reached similar levels to those presented by the HIV-neg group. Moreover, IP-10 was higher in the LcHI group (*P* < 0.0001) than the HIV-neg group (Fig. 3a). In the AHI group, cART was also able to reduce significantly (*P* = 0.0005) the IL-18 levels 6 months after cART initiation compared with pre-ART levels and reached similar levels to those observed in HIV-1 negative individuals (Fig. 3b). Despite the significant reduction of sCD163 levels after 6 months of cART in the EcHI group (*P* = 0.0186), but not in AHI, compared to the pre-ART levels, both groups presented levels similar to the HIV-1-uninfected group at this visit. This marker was higher in the LcHI group than in the HIV-neg group (*P* = 0.0060) (Fig. 3c). We observed higher sCD14 levels in the EcHI group after 6 months of cART than in the HIV-neg group (*P* = 0.0214) and the pre-ART point of the group (*P* = 0.0068) (Fig. 3d). No significant differences were observed in CRP and IL-6 among the studied groups (Additional file 1: Figure S1). We also evaluated IP-10 and IL-18 levels after 1, 2 and 3 months of cART initiation in the AHI group and observed that after 2 months, IP-10 and IL-18 reached levels that were not different from HIV-neg individuals (Additional file 1: Figure S2).

In analyses adjusted by age and group, at the pre-ART visit, the levels of IP-10 (*P* < 0.0001 for EcHI and AHI), IL-18 (*P* = 0.0005 for EcHI and *P* < 0.0001 for AHI) and sCD163 (*P* = 0.0001 for EcHI and *P* = 0.0056 for AHI) remained higher than those in the HIV-1-neg group. After adjustment, we found a slightly higher level of IL-6 in the AHI group than the HIV-1-neg group at the Pre-ART visit (*P* = 0.0418).

### CD8^+^ and CD4^+^ T cell activation reach levels that are comparable with HIV-1 uninfected individuals after 12 months of cART initiation during primary infection

**Fig. 4:**
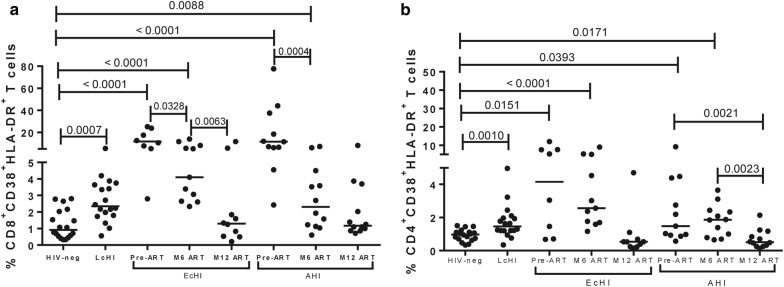
T cell activation on pre-ART and 6 months after. CD4^+^ (**a**) and CD8^+^ (**b**) T cell activation were measured by flow cytometry. *P* values were calculated using the Mann–Whitney test. All values < 0.05 were considered statistically significant. HIV-neg, HIV-1 uninfected individuals; LcHI, late treated chronic HIV-1 infection (median of 5 years after cART initiation); EcHI, early chronic HIV-1 infection; AHI, acute HIV-1 infection; M6 ART, 6 months after ART start; and M12 ART, 12 months after ART start

We observed a progressive decrease in CD8^+^ T cell activation after cART initiation in both groups of individuals recently infected with HIV-1 (Fig. 4a). A significant decline in the frequency of activated CD8^+^ T cells was observed comparing pre-ART and M6 ART visits in both groups. However, CD8^+^ T cell activation was similar to the HIV-neg group only after 12 months of cART. The CD4^+^ T cell activation, as observed to CD8^+^ T cells, only reached similar values to the observed in HIV-1-uninfected individuals after 12 months of cART. In the AHI group, we did not observe a decrease at M6, but after 12 months after cART the levels of CD4^+^ T cell activation were similar to the observed in HIV-neg group. In the analyses adjusted by age and group at the pre-ART visit, the frequency of activated CD8^+^ T cells remained higher than the HIV-neg group for both the EcHI (*P* = 0.0264) and AHI (*P* < 0.0001) groups, data not shown.

### IP-10 correlates with HIV-1 viral load at the pre-ART visit

**Fig. 5:**
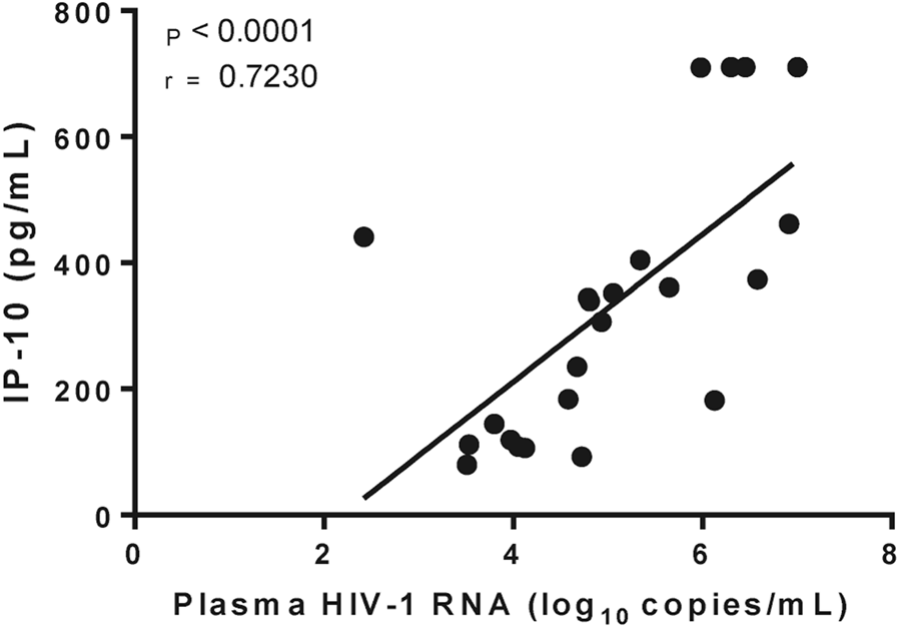
Correlation between IP-10 and HIV-1 plasmatic viral load. P and r values were calculated using Pearson correlation test. All values < 0.05 were considered as statistically significant

Among the PHI individuals (AHI and EcHI groups), viral loads showed a strong positive correlation with IP-10 at pre-ART visit (*P* = 0.0006 and r = 0.6610) (Fig. 5). In the adjusted analysis, this correlation remained significant (*P* = 0.0019), and we found a significant association between sCD14 and viral load (*P* = 0.0012) after age adjustment (Additional file 1: Table S2). No correlation was found between IL-18 or sCD163 and viral load among the PHI individuals. We also evaluated the total HIV-1 DNA in PBMCs of AHI individuals at the pre-ART visit and found no correlation with T cell activation or markers of inflammation.

### CD4/CD8 ratio correlates with inflammatory and activation markers

CD4/CD8 ratio have been pointed as a more accurate marker of immune dysfunction than absolute CD4^+^ T cell count [44]. Negative correlations between CD4/CD8 ratio and IP-10 (*P* = 0.0112 and r = − 0.5416), IL-18 (*P* = 0.0068 and r = − 0.5713) and CD8^+^ T cell activation (*P* = 0.0124 and r = − 0.6000) were also observed (Additional file 1: Fig S3). No correlation with sCD14, sCD163, IL-6 or CRP with CD4/CD8 ratio was observed.

## Discussion

This is the first study evaluating inflammation and T cell activation in Brazilian recent HIV-1 infected individuals. In this longitudinal study, we found that cART initiation in the HIV-1 acute infection had a higher impact on inflammation reduction and CD4^+^ T cell recovery than cART initiation in early chronic phase. IP-10, IL-18, sCD163, and CD8^+^ T cell activation were elevated in early infected individuals from a Rio de Janeiro, Brazil when compared with the HIV-neg group. However, among the inflammatory markers, only IP-10 presented a positive correlation with the HIV-1 viral load.

There is clear evidence of the benefits of the early initiation of cART [2–7], and the WHO currently recommends the immediate cART initiation in individuals with a positive HIV-1 diagnosis, which is a recommendation followed by Brazil since 2013 [45]. The primary goals of this strategy are the following: to preserve the immune system, to avoid the early development of AIDS and non-AIDS related diseases and to reduce HIV-1 transmission. Although the CD4^+^ T cell count is no longer a criteria to define the moment of cART initiation, it is still a valuable marker of immune reconstitution in individuals starting cART and is also routinely used in clinical follow-up in settings were viral load monitoring is poor. Here, we observed an earlier normalization of CD4^+^ T cell counts in the group that started cART during the Fiebigs I-V acute phase (AHI). After 12 months of cART, the group that started cART only in the early chronic phase remained with lower CD4^+^ T cell counts than the HIV-neg group. Studies have explored the CD4/CD8 ratio as a marker of persistent immune activation and inflammation, even after long-term suppressive cART [46–48]. A large study conducted with more than 400 MSM showed that 56% inverted the CD4/CD8 ratio around 3 months after seroconversion [49]. In contrast with the normalization of CD4^+^ T cell counts in the AHI group after 6 months of cART, we observed a lower CD4/CD8 ratio in both the AHI and EcHI groups than in the HIV-neg group even at 12 months of cART. A previous study demonstrated that an altered CD4/CD8 ratio remains after 2 years of ART in individuals who started ART in acute infection, even during the Fiebig I stage [4]. In our study, the inverted CD4/CD8 ratio is mainly explained by the high CD8^+^ T cell counts (data not shown). Once cART suppressed viral loads after 6 months of treatment, it is probably that other factors, possibly the persistent inflammation and immune activation, than only the viral loads, affect the CD8^+^ T cell compartment. Indeed, we found negative correlations between the CD4/CD8 ratio and IP-10, IL-18, and CD8^+^ T cell activation. Microbial translocation also correlated with CD8^+^ T cell counts [50], and in our study, we found no higher levels of sCD14, a monocyte activation marker also used as a microbial translocation marker [51], in both AHI and EcHI groups when compared to the HIV-neg group at the pre-ART visit. An unexpected increase in sCD14 at M6 was observed in EcHI group, which could be explained by the later cART initiation in this group compared with AHI, allowing a more extensive damage in the gut mucosa. In contrast, we did not observe a higher level of this marker in LcHI group, composed by individuals with more than 5 years in cART, compared to HIV-neg group, this could indicate that a longer time of cART is necessary to decrease sCD14 to similar levels to the observed in HIV-neg individuals. A previous study [5] demonstrated that sCD14 levels remained higher in individuals treated during acute HIV infection than in HIV-uninfected individuals after 2 years, even though this group, after 12 months of treatment, presented lower sCD14 levels compared to individuals who started cART in the chronic phase.

Early cART initiation reduces inflammation and immune activation [3, 4, 6, 7]. IP-10 was one of the markers of inflammation elevated in a pre-ART visit in AHI and EcHI individuals, significantly reducing after 6 months of cART in both groups, although EcHI individuals did not achieve normal values. This marker was associated with an increased risk of HIV-1 acquisition, faster disease progression, and high plasmatic viral loads [31, 52]. IP-10 binding CXCR3 and CD4^+^CXCR3^+^ T cells are the main targets of HIV-1 infection. Thus, it is likely that a high level of IP-10 contributes to the elevated viral replication by attracting target cells that subsequently induces further IP-10 production [53]. After 2 months of treatment the levels of IP-10 in AHI group reached similar levels to the observed in HIV-1 neg-group. At this time, most individuals presented undetectable or very low (< 100 copies/mL) viral load, except for one individual (1311 copies/mL), who presented pre-ART viral load > 10 million copies/mL. We also found a high level of IL-18 at the pre-ART visit in this group. After 2 months of cART, these levels in the AHI group were similar to that observed in the HIV-neg group, following the same dynamics observed for IP-10. IL-18 is produced in response to inflammasome activation, as reviewed by Dinarello et al. 2013 [54]. The inflammasome is activated in response to several stimuli, including LPS, and HIV-1 can activate inflammasome [55, 56]. The comparison of our results of IP-10 and IL18 levels with other studies is limited by variations in assay and characteristics of study population, as age and coinfections. However, in a general way, we founded studies which showed higher [3, 6, 30, 57], lower [43], and similar [31] values of IP-10, and a similar level of IL-18 when compared with a French study [30].

Studies have demonstrated a decrease in T cell activation, mainly CD8^+^ T cell activation, after cART initiation during the acute phase of HIV-1 infection [3, 35]. Here, we observed an earlier decrease in markers of inflammation than in T cell activation. However, we detected an earlier normalization of CD8^+^ T cell activation after cART initiation than that reported by two previous studies, which demonstrated higher levels of this marker, even after 12 [7] or 24 months [35], than those observed in HIV-1-uninfected individuals. In both studies, the median of CD8^+^ T cell activation was higher than that observed here, which could explain the longer time required for CD8^+^ T cell activation normalization. Moreover, as observed to markers of inflammation, other factors, as coinfections, may also explain the differences.

The HIV-1 subtype B was the prevalent in our study groups of recent infected individuals, followed by B/C and B/F recombinants. When we compared HIV-1 subtype B versus non-B individuals, we founded no differences in inflammation or T cell activation markers. One individual from EcHI group was infected by HIV-1 subtype D, which have been associated with a faster disease progression [58]. However, this individual presented viral load and CD4^+^ T cell count comparable with group median, and the same was observed to markers of inflammation and T cell activation.

Our study has some limitations that should be highlighted. The first limitation is the small size of our cohort. Indeed, the identification of individuals in the acute or early phase of HIV-1 infection is a major challenge. Second, our groups of recently infected individuals were composed exclusively by MSM, different from the LcHI and HIV-neg groups. Moreover, we only quantified the total HIV-1 DNA levels, with no differentiation between 2-LTR and integrated DNA and no functional assay was performed to test the size of the competent reservoir. A longer follow-up certainly will help to achieve a better understanding of the dynamics of immune activation, inflammation, and immune reconstitution after the early cART initiation.

Alltogether, our results reinforce that early cART initiation during HIV infection can reduce systemic inflammation, but the earlier normalization of the markers related to this phenomenon was only observed when cART is initiated in the acute phase. We suggest that IP-10 was the best marker to evaluate inflammation on recent HIV-1 infection, once it was elevated at pre-ART visit and showed a positive correlation with plasmatic viral load. CD8^+^ T cell activation presented a different reduction dynamic when compared to IP-10 and IL-18, and it only reached normal levels one year after cART initiation.

## Additional file

**Additional file 1: Figure S1.** Plasmatic markers of inflammation. CRP (**a**) and IL-6 (**b**) were measured by ELISA. *P* values were calculated using the Mann–Whitney test. All values < 0.05 were considered statistically significant. HIV-neg, HIV-1 uninfected individuals; LcHI, late chronic HIV-1 infection; EcHI, early chronic HIV-1 infection; AHI, acute HIV-1 infection; M6 ART, 6 months after cART start after cART start. **Figure S2.** Decreasing dynamics of inflammatory markers among acute treated HIV infected individuals. IP-10 (**a**) and IL-18 (**b**) were measured by ELISA at different time points after cART start. *P* value were calculated using the Mann–Whitney test. All values < 0.05 were considered statistically significant. HIV-neg, HIV-1 uninfected individuals. **Figure S3.** Correlations between markers of inflammation activation with CD4/CD8 ratio at pre-ART visit. IP-10 and CD4/CD8 ratio (**a**), IL-18 and HIV-1 CD4/CD8 ratio (**b**), CD8 activation and CD4/CD8 ratio (**c**). *P* and *r* values were calculated using the Spearman test. All values < 0.05 were considered statistically significant. **Table S1.** HIV-1 subtypes. **Table S2.** Age adjusted analyzes of the association between sCD14 levels and HIV-1 viral load at pre-ART visit.
