# Supplementary material for: Reduction of inflammation and T cell activation after 6 months of cART initiation during acute, but not in early chronic HIV-1 infection
Source: Retrovirology. 2018 Dec 12;15:76. doi: 10.1186/s12977-018-0458-6 (PMC6291985; doi:10.1186/s12977-018-0458-6)
Supplement: Supplementary file 1 — Additional file 1: Figure S1. Plasmatic markers of inflammation. CRP (a) and IL-6 (b) were measured by ELISA. P values were calculated using the Mann–Whitney test. All values < 0.05 were considered statistically significant. HIV-neg, HIV-1 uninfected individuals; LcHI, late chronic HIV-1 infection; EcHI, early chronic HIV-1 infection; AHI, acute HIV-1 infection; M6 ART, 6 months after cART start after cART start. Figure S2. Decreasing dynamics of inflammatory markers among acute treated HIV infected individuals. IP-10 (a) and IL-18 (b) were measured by ELISA at different time points after cART start. P value were calculated using the Mann–Whitney test. All values < 0.05 were considered statistically significant. HIV-neg, HIV-1 uninfected individuals. Figure S3. Correlations between markers of inflammation activation with CD4/CD8 ratio at pre-ART visit. IP-10 and CD4/CD8 ratio (a), IL-18 and HIV-1 CD4/CD8 ratio (b), CD8 activation and CD4/CD8 ratio (c). P and r values were calculated using the Spearman test. All values < 0.05 were considered statistically significant. Table S1. HIV-1 subtypes. Table S2. Age adjusted analyzes of the association between sCD14 levels and HIV-1 viral load at pre-ART visit. [file 12977_2018_458_MOESM1_ESM.docx]

A

B

**FIGURE S1. Plasmatic markers of inflammation.** CRP (A) and IL-6 (B) were measured by ELISA. P values were calculated using the Mann-Whitney test. All values < 0.05 were considered statistically significant. HIV-neg, HIV-1 uninfected individuals; LcHI, late chronic HIV-1 infection; EcHI, early chronic HIV-1 infection; AHI, acute HIV-1 infection; M6 ART, six months after cART start after cART start.

A

B

**FIGURE S2. Decreasing dynamics of inflammatory markers among acute treated HIV infected individuals.** IP-10 (A) and IL-18 (B) were measured by ELISA at different time points after cART start. P value were calculated using the Mann-Whitney test. All values < 0.05 were considered statistically significant. HIV-neg, HIV-1 uninfected individuals.

A

B

C

**FIGURE S3. Correlations between markers of inflammation activation with CD4/CD8 ratio at pre-ART visit.** IP-10 and CD4/CD8 ratio (A), IL-18 and HIV-1 CD4/CD8 ratio (B), CD8 activation and CD4/CD8 ratio (C). P and r values were calculated using the Spearman test. All values < 0.05 were considered statistically significant.

**Table S1:** HIV-1 subtypes.

| Subtype | EcHI (n = 11) | AHI (n = 12) |
| --- | --- | --- |
| B | 7 | 6 |
| B/C | 1 | 4 |
| F or B/F | 1 | 2 |
| Other | 2 | 0 |

**Table S2:** Age adjusted analyzes of the association between sCD14 levels and HIV-1 viral load at pre-ART visit.

|  | Estimate | Std. Error | t value | P value |
| --- | --- | --- | --- | --- |
| (Intercept) | 430.795 | 446.033 | 0.966 | 0.34566 |
| Age | -2.161 | 12.762 | -0.169 | 0.86722 |
| Viral load (log) pre-ART | 250.524 | 66.606 | 3.761 | 0.00123 |

Linear regression models
